# Higher efficacy of anti-IL-6/IL-21 combination therapy compared to monotherapy in the induction phase of Th17-driven experimental arthritis

**DOI:** 10.1371/journal.pone.0171757

**Published:** 2017-02-03

**Authors:** Debbie M. Roeleveld, Renoud J. Marijnissen, Birgitte Walgreen, Monique M. Helsen, Liduine van den Bersselaar, Fons A. van de Loo, Peter L. van Lent, Peter M. van der Kraan, Wim B. van den Berg, Marije I. Koenders

**Affiliations:** Department of Experimental Rheumatology, Radboud University Medical Center, Nijmegen, the Netherlands; Universitatsklinikum Jena, GERMANY

## Abstract

Th17 cells and their cytokines are linked to the pathogenesis of rheumatoid arthritis, a chronic autoimmune disease characterized by joint inflammation. Th17 development is initiated by combined signaling of TGF-β and IL-6 or IL-21, and can be reduced in the absence of either IL-6 or IL-21. The aim of this study was to assess whether combinatorial IL-6/IL-21 blockade would more potently inhibit Th17 development, and be more efficacious in treating arthritis than targeting either cytokine. We assessed *in vitro* Th17 differentiation efficacy in the absence of IL-6 and/or IL-21. To investigate *in vivo* effects of IL-6/IL-21 blockade on Th17 and arthritis development, antigen-induced arthritis (AIA) was induced in IL-6^-/-^ x IL-21R^-/-^ mice. The therapeutic potential of this combined blocking strategy was assessed by treating mice with collagen-induced arthritis (CIA) with anti-IL-6R antibodies and soluble (s)IL-21R.Fc. We demonstrated that combined IL-6/IL-21 blocking synergistically reduced *in vitro* Th17 differentiation. In mice with AIA, absence of IL-6 and IL-21 signaling more strongly reduced Th17 levels and resulted in stronger suppression of arthritis than the absence of either cytokine. Additionally, anti-IL-6/anti-IL-21 treatment of CIA mice during the arthritis induction phase reduced disease development more potent than IL-6 or IL-21 inhibition alone, as effective as anti-TNF treatment. Collectively, these results suggest dual IL-6/IL-21 inhibition may be a more efficacious therapeutic strategy compared to single cytokine blockade to suppress arthritis development.

## Introduction

Rheumatoid arthritis (RA) is a chronic autoimmune disease that affects up to 1% of the population worldwide. It is characterized by chronic inflammation leading to destruction of bone and cartilage of synovial joints [1]. Although the use of biologicals blocking a specific pathogenic target considerably improved symptoms and prognosis of some RA patients, approximately 30% of the patients still fail to respond adequately to currently available treatment [2–5]. Interestingly, anti-TNFα non-responders were shown to have increased levels of T helper (Th)17 cells in their circulation [5–7], suggesting Th17 cells and Th17-derived cytokines might be interesting alternative therapeutic targets in RA. Th17 cells produce various pro-inflammatory cytokines like IL-17, IL-21, and IL-22, that have been linked to RA pathogenesis [8–14]. Arthritis animal models have further elucidated the role of these Th17 cytokines in the processes of joint inflammation and destruction of cartilage and bone [15,16]. These findings indicate that Th17 cells and their cytokines are important contributors to the pathogenesis of chronic and destructive joint inflammation during RA.

Differentiation of Th17 cells from naïve CD4+ T cells is mediated by the enhanced expression of the transcription factor RAR-related orphan nuclear receptor γt (RORγt), originally thought to be induced exclusively by the combined signaling of TGF-β and IL-6 [17,18]. However, later it was shown that signaling of TGF-β combined with the autocrine signaling of IL-21 could drive Th17 differentiation as well [19–21]. Both IL-6 and IL-21 induce upregulation of the IL-23 receptor (IL-23R) in naïve CD4+ T cells, making those cells responsive to IL-23. IL-23 can subsequently amplify the differentiation of Th17 cells [20,22]. When either the IL-6 or IL-21 signaling pathway is absent, differentiation of naïve T cells towards Th17 cells is reduced. However, to date it is not known whether Th17 differentiation can be further reduced when both IL-6 and IL-21 pathways are blocked. Therefore, the purpose of this study was to determine whether IL-6/IL-21 combinatorial pathway blockade has additional inhibitory effects on Th17 differentiation, and more effectively reduces development of T cell-dependent experimental arthritis. Firstly, we assessed the effects of complete IL-6/IL-21 pathway inhibition on Th17 differentiation *in vitro*. Secondly, we used IL-6^-/-^ x IL-21R^-/-^ mice to study the *in vivo* effects of cytokine pathway blockade on Th17 differentiation and development of experimental arthritis. Finally, we investigated the therapeutic potential of our combined blocking strategy by treating mice with collagen-induced arthritis (CIA) with anti-IL-6R antibodies and soluble (s)IL-21R.Fc.

## Materials and methods

### Mice

Wild type (WT) C57Bl6/J mice and DBA-1 mice were purchased from Janvier-Elevage (Le Genest Saint Isle, France), and breeding pairs of the IL-21R-deficient mice on a C57Bl6/J background were provided by Pfizer. Breeding pairs of the IL-6^−/−^ mice, back-crossed eight times with C57Bl6/J, were a kind gift from Dr Manfred Kopf (Basel, Switzerland)[23]. Animals were used between 10 and 12 weeks of age and were housed in filter-top cages under specific pathogen-free conditions. Microbiome synchronization was promoted by housing purchased WT mice near the genetically modified mice for a period of at least one week, before initiating experiments. A standard diet and water were provided ad libitum. Before being sacrificed by cervical dislocation, mice were anesthetized using 2–3% isoflurane. All animal procedures were approved by the ethics committee of the Radboud University Nijmegen (approval numbers 2014–045, 2010–002, and 2012–207).

### *In vitro* stimulation of CD4+ T cells

Naïve murine T helper cells were negatively selected from spleen, isolated from IL-21R^-/-^ or WT C57Bl6/J mice using a mouse naïve CD4^+^ T Cell Isolation Kit (Miltenyi Biotec, Bergisch Gladbach, Germany). Naïve CD4^+^ T cells were cultured for four days in 6-wells plates at 37°C, 5% CO_2_, in RPMI-1640 medium containing 5% fetal calf serum (FCS), 50 μm β-mercaptoethanol, 50 μg/ml gentamycin, and 1% pyruvate. The following Th17 stimulation cocktail was added, with or without recombinant mouse (rm)IL-6 (50 ng/ml; eBioscience, San Diego, CA, USA): anti-CD3 (5 μg/ml; eBioscience; adsorbed to the wells over night at 4°C), anti-CD28 (2.5 μg/ml; eBioscience), anti-IL-2 (5 μg/ml; eBioscience), TGF-β (1 ng/ml; eBioscience), IL-1β (10 ng/ml; kind gift of Pfizer), and TNFα (10 ng/ml; eBioscience). Differentiation efficacy was determined with flow cytometry. Supernatant cytokine levels were determined using the Luminex multianalyte technology, in combination with Milliplex cytokine kits (Merck Millipore, Darmstadt, Germany).

### *In vivo* study protocol I: Antigen-induced arthritis (AIA) in knockout mice

AIA was induced in WT, IL-21R^-/-^, IL-6^-/-^, and IL-21R^-/-^ x IL-6^-/-^ mice as previously described [24]. Inflammation of the knee joint was measured by ^99m^Technetium (^99m^Tc) pertechnetate uptake. Joint swelling was scored as the ratio of ^99m^Tc uptake of the arthritic right (R) and control left (L) knee joint. Joint swelling of the right knee joint was indicated as R:L ratios >1.1. Mice were sacrificed by cervical dislocation either at day 2 or day 7 after arthritis induction, and serum and joints were collected for further analysis. Draining lymph nodes (dLN) of mice sacrificed two days after arthritis induction were isolated to determine Th17 levels.

### *In vivo* study protocol II: Antibody treatment during collagen-induced arthritis (CIA)

CIA was induced as described previously [25]. Arthritis development was macroscopically scored on a scale of 0–2 per paw, according to changes in redness and/or swelling of the paws. IL-6 signaling was inhibited with a single bolus intraperitoneal (i.p.) injection with 8 mg of a rat anti-mouse IL-6 receptor antibody (MR16-1; kind gift of Chugai Pharmaceutical Co. Ltd). IL-21 was neutralized by three i.p. injections per week with 200 μg soluble (s)IL-21R.Fc (Pfizer, New York, NY, USA), from now on referred to as anti-IL-21 therapy [26,27]. Treatment was initiated at the day of immunization (day 0, early treatment), or at the day of booster injection (day 21, late treatment). Both treatments were administered as single and combination treatments. The TNFα inhibitor Enbrel (200 μg; Pfizer) and rat IgG1 (200 μg; Pfizer) were injected as positive and negative control respectively, both i.p. 3 times a week. Mice were terminally bled at day 35 to determine serum antibody levels. Subsequently ankle joints were isolated for X-ray and histological analysis, and draining popliteal and inguinal lymph nodes were isolated to determine T cell levels.

### Histology

Isolated joints were fixed for at least four days in 4% formaldehyde, decalcified in 5% formic acid, dehydrated, and embedded in paraffin. Standard frontal sections of 7 μm were mounted on SuperFrost slides (Menzel-Gläser, Braunschweig, Germany), and stained with haematoxylin and eosin (H&E), or Safranin O (SO). Joint arthritis severity was scored on an arbitrary scale of 0–3, as previously described [28]. Histopathological changes were scored on three semiserial sections of the joint, in a blindfolded manner.

### Flow cytometry

Cells were stimulated four hours with phorbol myristate acetate (PMA; 50 ng/ml; Sigma-Aldrich, Saint Louis, MO, USA), ionomycin (1 μg/ml; Sigma-Aldrich) and the Golgi-traffic inhibitor Brefeldin (1 μl/ml; BD Biosciences, Franklin Lakes, NJ, USA). Differentiated T cells or isolated LN cells were stained with anti-CD3-PE (BD Biosciences) and anti-CD4-APC (Biolegend, San Diego, CA, USA), fixed and permeabilized using BD Cytofix/Cytoperm (BD Biosciences), followed by intracellular staining with anti-IL-17-FITC (Biolegend), anti-IFNγ-FITC (BD Biosciences), or appropriate isotype-matched control antibodies (all from BD Biosciences). Cells were measured on a FACSCalibur using the CellQuest software (BD Biosciences), and analyzed using FlowJo software (version 7.6.5).

### Measurement of anti-mBSA and anti-CII antibodies

Levels of anti-mBSA and anti-CII antibodies were determined in serum using enzyme-linked immunosorbent assay (ELISA). In short, 10 ng of mBSA or 100 ng of bovine type II collagen were coated onto 96-well plates overnight. Nonspecific binding sites were blocked with 1% BSA in PBS-Tween (0.05%) for anti-mBSA antibody detection, and with a 5% milk powder solution for anti-CII antibody detection. Serial dilutions of mouse sera were incubated for 1 hour, before adding isotype-specific horseradish peroxidase-labeled goat anti-mouse Ig (1:1000). After incubation of another hour, 5-aminosalicyclic acid was added as a substrate, absorbance was subsequently measured at 450 nm.

### Statistics

To determine the level of statistical significance between means of experimental groups, the Kruskal-Wallis with a Dunns post-test, or a one-way ANOVA with a Bonferroni post-test was applied as indicated, using GraphPad Prism version 5. *P* values less than 0.05 are considered significant. Results are expressed as Tukey box plots.

## Results

### Combined blocking of IL-6 and IL-21 synergistically inhibits *in vitro* Th17 differentiation

**Fig 1:**
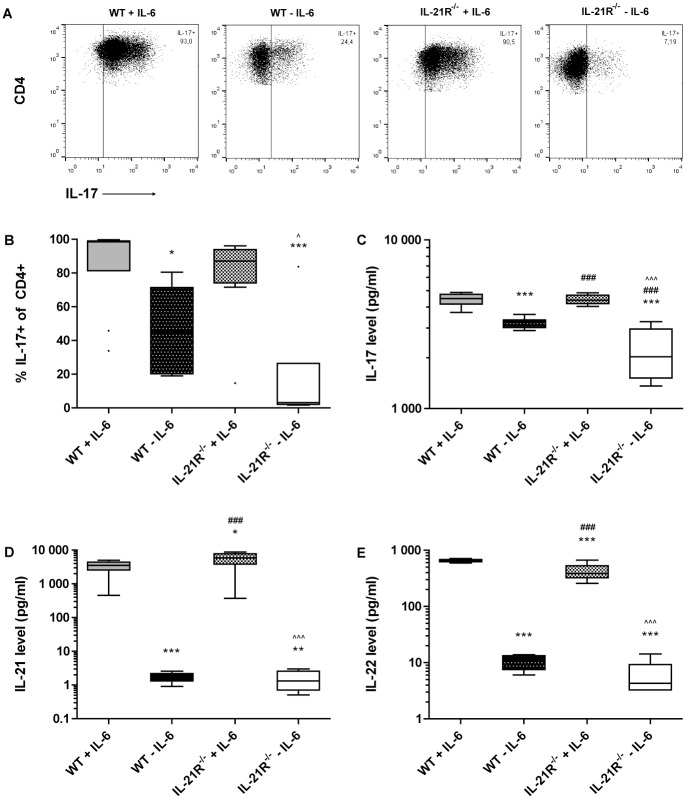
Highly reduced Th17 differentiation in the absence of both IL-6 and IL-21 signaling. WT and IL-21R^-/-^ naïve T cells were stimulated for four days with a differentiation cocktail either with or without IL-6 as described in *Methods*. Representative flow cytometry dot plots reflecting the IL-17+ fraction of the CD4+ population. Gates were set at a maximum of 0.3% IL-17-positive cells in the ‘fluorescence minus one’ control per condition, without addition of IL-17A antibodies (A). Summary of the relative proportion of IL-17+ cells within the CD4+ population (B). Culture supernatant levels of IL-17 (C), IL-21 (D), and IL-22 (E). 6–10 mice/group; *p<0.05, **p<0.01, ***p<0.001 versus WT + IL-6; ^###^p<0.001 versus WT—IL-6; ^p<0.05, ^^^p<0.001 versus IL-21R^-/-^ + IL-6; A—Kruskal-Wallis, B-D—One-way ANOVA.

To investigate the exchangeability of IL-6 and IL-21 and potential additive or synergistic effects of these cytokines during Th17 differentiation, we first determined the differentiation efficacy of naïve T cells towards Th17 cells in the presence of both IL-6 and endogenous IL-21. After culturing, 86% of CD4+ cells were positive for IL-17 (Fig 1A and 1B), and produced high levels of IL-17 (Fig 1C). Subsequently, we determined Th17 differentiation efficacy in the absence of either IL-6 or IL-21 signaling. Without IL-6, naïve T cells reached only 46% of Th17 development upon stimulation, while Th17 differentiation efficacy of naïve IL-21R^-/-^ T cells was similar to that observed after stimulation of WT cells (Fig 1A and 1B). Interestingly, when both IL-6 and IL-21 signaling pathways were absent, only 17% of CD4+ cells differentiated into Th17 cells after culturing (Fig 1A and 1B), and IL-17 secretion was reduced by 50% (Fig 1C). High endogenous IL-21 levels were detected in supernatants of cultures with IL-6 (Fig 1D), potentially contributing to Th17 development in an autocrine fashion [19–21]. Finally, secretion of IL-22 in supernatant of those cultures was confirmed (Fig 1E). This shows that by blocking IL-6 and IL-21 signaling pathways *in vitro*, Th17 differentiation is dramatically reduced, thereby providing excellent rationale for further *in vivo* studies.

### Th17 differentiation and arthritis severity are potently reduced by combinatorial blockade of IL-6 and IL-21 signaling pathways

**Fig 2:**
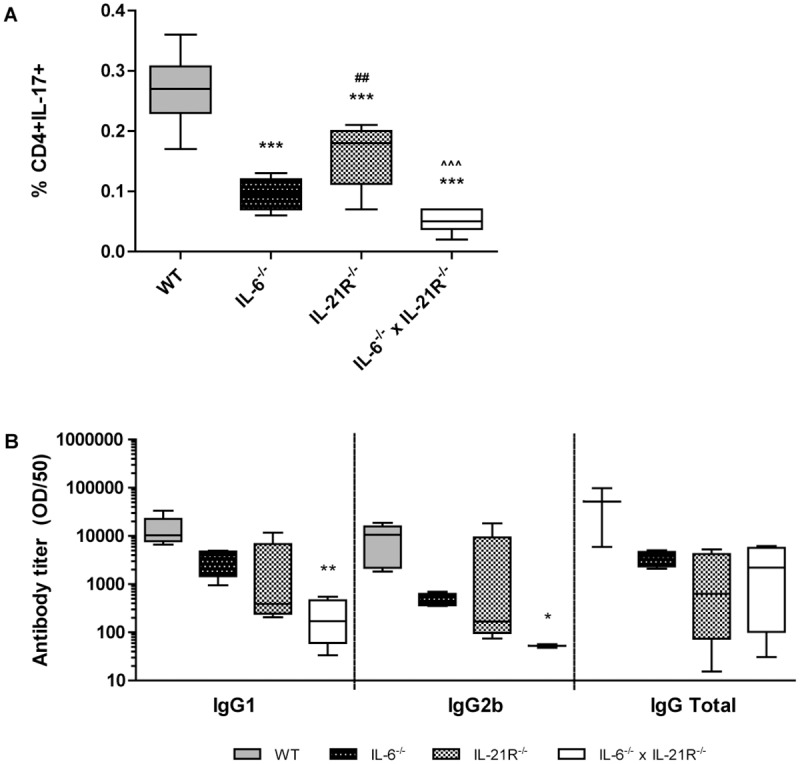
IL-6 and IL-21 play an important role during in vivo Th17 differentiation and antibody production. AIA was induced in WT, IL-6^-/-^, IL-21R^-/-^, and IL-6^-/-^ x IL-21R^-/-^ mice. Draining lymph node CD4+IL-17+ cell fraction two days after arthritis induction as measured using flow cytometry (A; n = 10/group). Serum IgG1, IgG2b, and total IgG levels as measured by ELISA (B; n = 5/group). *p<0.05, **p<0.01, ***p<0.001 versus WT; ^##^p<0.01 versus IL-6^-/-^; ^^^p<0.001 versus IL-21R^-/-^; A—One-way ANOVA, B—Kruskal-Wallis.

We next investigated the *in vivo* effects of IL-6 and IL-21 pathway blockade on Th17 differentiation and the development of experimental arthritis. The T cell-driven AIA model was induced in WT, IL-6^-/-^, IL-21R^-/-^, and IL-6^-/-^ x IL-21R^-/-^ mice, and the number of Th17 cells in draining LNs was assessed two days after arthritis onset. Expression of various T (CD3, CD4, CD8) and B (CD45R, CD19, CD22) cell markers was unaffected in spleens of IL-6^-/-^ x IL-21R^-/-^ mice as compared to WT, indicating normal lymphocyte development (data not shown). Among total measured cell population of WT mice, 0.3% was CD4+IL-17+ (Fig 2A). In line with our *in vitro* data, Th17 levels were reduced in mice lacking IL-6 signaling, and in mice in which both IL-6 and IL-21R were absent. Remarkably, in contrast to our *in vitro* data (Fig 1), IL-21R^-/-^ mice also showed significantly reduced Th17 numbers *in vivo* (Fig 2A). In addition to its importance in T cell development, IL-21 has been implicated in B cell development and antibody production [29,30], processes relevant to RA and important in this experimental arthritis model as well. Hence, we were interested in determining the effect of a lack in IL-6 but especially in IL-21 on antibody production in AIA. Unexpectedly, we did not observe any effect on antibody titers in mice deficient only for the IL-21R (Fig 2B). However, mice lacking both IL-6 and IL-21R expression showed reduced antibody production as compared to WT controls (Fig 2B), suggesting that combined deficiency of IL-6 and IL-21 can significantly suppress the development of disease-relevant antibodies.

**Fig 3:**
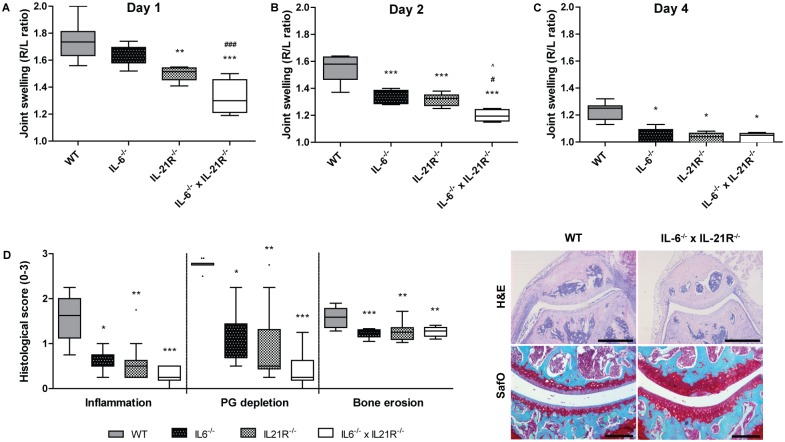
Antigen-induced arthritis severity is potently reduced by combinatorial blockade of IL-6 and IL-21 signaling pathways. Joint swelling of WT, IL-6^-/-^, IL-21R^-/-^, and IL-6^-/-^ x IL-21R^-/-^ mice at day 1 (A), day 2 (B), and day 4 (C) after arthritis induction, depicted as ratio between right and left knee joint, measured by ^99m^Technetium pertechnetate uptake in the joint (n = 6/group). Histologically scored inflammation, bone erosion (both H&E staining, scale bar 500 μM), and cartilage proteoglycan depletion (SafO staining, scale bar 200 μM) (D; n = 10/group). *p<0.05, **p<0.01, ***p<0.001 versus WT; ^#^p<0.05, ^###^p<0.001 versus IL-6^-/-^; ^p<0.05 versus IL-21R^-/-^; A+B One-way ANOVA, C+D Kruskal-Wallis.

In line with lowered Th17 levels, reduced joint swelling was measured in the absence of IL-6 or IL-21R, as compared to WT controls (Fig 3A–3C). Interestingly, joint swelling was reduced more potently in IL-6^-/-^ x IL-21R^-/-^ mice compared to mice lacking either IL-6 or IL-21R expression early in disease development (Fig 3A and 3B). Even though joint swelling of WT mice started to decline four days after arthritis induction, joint swelling was still significantly lower in all knock out groups (Fig 3C). Importantly, when assessing histologically scored inflammation, cartilage proteoglycan (PG) depletion, and bone erosion at this time point, we observed significantly reduced damage in IL-6^-/-^, IL-21R^-/-^, and IL-6^-/-^ x IL-21R^-/-^ mice as compared to the WT controls for all parameters (Fig 3D).

Overall, this study confirmed the importance of IL-6 and IL-21 during Th17 development *in vivo*, and showed higher potency of blocking both cytokine pathways above blocking either one in reducing the severity of arthritis.

### Combination therapy with anti-IL-6R antibodies and sIL-21R.Fc more potently suppresses disease development than targeting either cytokine during the induction phase of experimental arthritis

To determine the therapeutic potential of combined IL-6/IL-21 signaling pathway blockade during experimental arthritis, mice with CIA were treated with anti-IL-6R antibodies and/or sIL-21R.Fc starting either from the day of immunization (day 0, early treatment), or from the day of the booster injection (day 21, late treatment).

**Fig 4:**
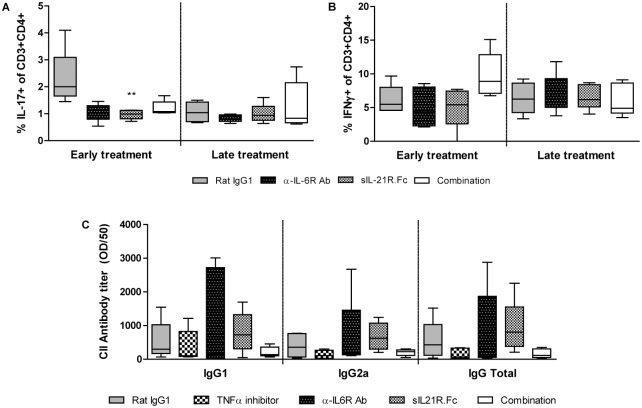
Effect of anti-IL-6 and/or anti-IL-21 treatment on Th17, Th1, and antibody development in CIA mice. Th17 (A) and Th1 (B) levels in draining lymph nodes as measured using flow cytometry, and anti-CII antibody level in serum (C) as measured by ELISA of mice with CIA receiving anti-IL-6 and/or anti-IL-21 treatment. n = 5/group; **p<0.01 versus Rat IgG1; A+C—Kruskal-Wallis, B—One-way ANOVA.

Surprisingly, mice receiving early anti-IL-6R antibody and/or anti-IL-21 treatment, only showed a minor trend towards lower Th17 levels in their LNs as compared to isotype control mice (Fig 4A). This trend in Th17 reduction was not present when treatment was initiated later in the disease process, i.e. at the day of booster injection. Th1 levels were largely unaffected, except for a minor trend towards an increase in mice receiving early combination treatment (Fig 4B). In contrast to observations in our AIA study using knockout mice (Fig 2B), we did not observe any changes in antibody profile when using this anti-IL-6/anti-IL-21 treatment strategy in mice with CIA (Fig 4C).

**Fig 5:**
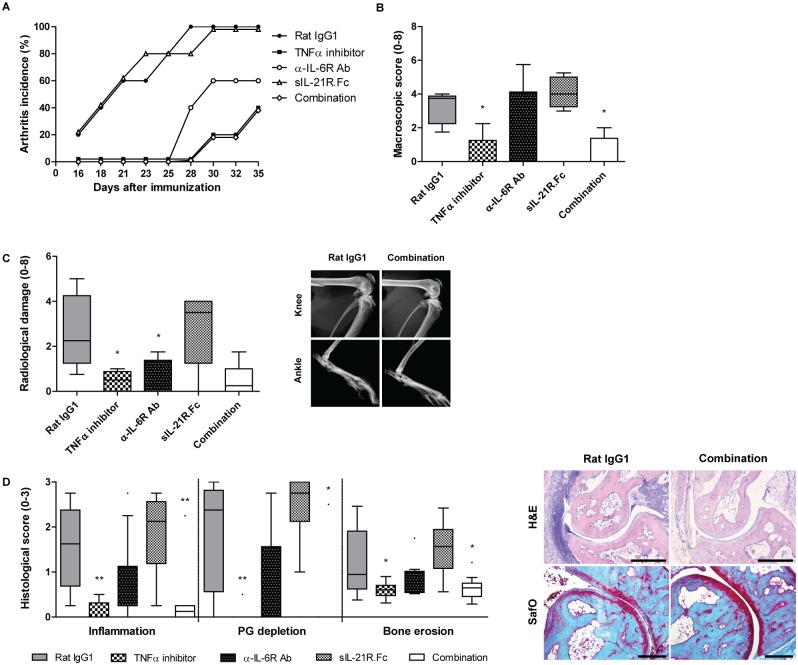
Arthritis incidence and severity of mice receiving anti-IL-6 and/or anti-IL-21 treatment during the induction phase of the disease. Collagen-induced arthritis was initiated in DBA-1 mice, subsequently treated with anti-IL-6R antibodies and/or sIL-21R.Fc from the day of the immunization injection (d = 0; n = 5/group). Arthritis incidence (A) and severity (B) based on macroscopic scoring. Bone damage as measured using the Faxitron depicted as radiological damage (C). Histologically scored inflammation, bone erosion (both H&E staining, scale bar 500 μM), and cartilage proteoglycan depletion (SafO staining, scale bar 200 μM) (D). *p<0.05, **p<0.01 versus Rat IgG1; One-way ANOVA.

**Fig 6:**
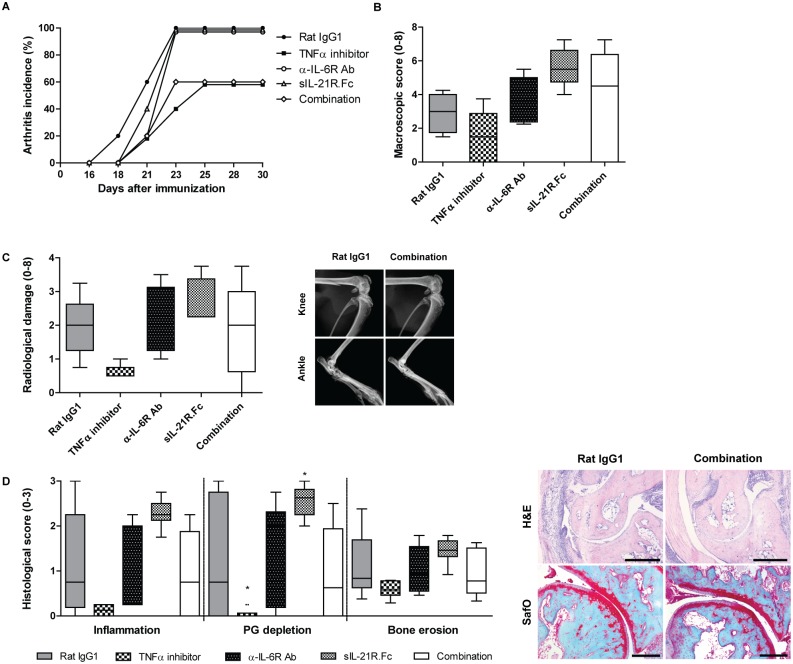
Arthritis incidence and severity of mice receiving anti-IL-6 and/or anti-IL-21 treatment late in disease development. Collagen induced arthritis was initiated in DBA-1 mice, subsequently treated with anti-IL-6R antibodies and/or sIL-21R.Fc from the day of booster injection (d = 21; n = 5/group). Arthritis incidence (A) and severity (B) based on macroscopic scoring. Bone damage as measured using the Faxitron depicted as radiological damage (C). Histologically scored inflammation, bone erosion (both H&E staining, scale bar 500 μM), and cartilage proteoglycan depletion (SafO staining, scale bar 200 μM) (D). *p<0.05 versus Rat IgG1; One-way ANOVA.

Even though the effect of cytokine neutralization on Th17 levels was minor, we observed potent clinical effects of this treatment strategy. Mice receiving early αIL-6R antibody treatment showed potently reduced disease incidence compared to isotype-treated mice, with only 60% of these mice having arthritis at day 35 (Fig 5A). Interestingly, arthritis incidence was further reduced when mice were treated with the anti-IL-6R/anti-IL-21 combination therapy, resulting in only 40% of mice developing arthritis. This greatly reduced disease incidence shows the potential of the early IL-6/IL-21 combination strategy over single cytokine inhibition in blocking arthritis development, with comparable efficacy as the positive control group receiving TNFα inhibitors. In contrast to early anti-IL-6R therapy, late treatment with anti-IL-6R antibodies could not inhibit arthritis; all mice developed arthritis already around day 23 (Fig 6A). Remarkably, mice treated with the late anti-IL-6R/anti-IL-21 combination therapy show a clear protective effect on arthritis incidence; only 60% of those mice developed arthritis before the end of the study (day 30). Unfortunately, none of the late experimental treatments had an effect on arthritis severity, as joint swelling (Fig 6B), radiological bone damage (Fig 6C), and histological damage (Fig 6D), were all comparable to levels measured in the isotype control group. In mice treated from the day of immunization with anti-IL-6 and anti-IL-21 treatment on the other hand, clinical arthritis severity based on swelling and redness of the four paws was greatly reduced in comparison with the isotype control group (Fig 5B). Unexpectedly, we found reduced radiological scored bone damage only to be significant in early anti-IL-6R antibody receiving mice, with no additional value of blocking IL-21 (Fig 5C). Interestingly, histological damage was reduced more potent in mice receiving anti-IL-6/IL-21 combinatorial treatment than in mice receiving single anti-IL-6 or anti-IL-21 treatment (Fig 5D), altogether suggesting higher efficacy of this combination treatment strategy over single cytokine blockade during the induction phase of the disease.

## Discussion

IL-6 and IL-21 are important mediators in the differentiation of Th17 cells [17–19]. The redundancy and possible additive or synergistic effects of the IL-6 and IL-21 pathways, as well as the therapeutic potential of combined pathway blockade in Th17-driven autoimmunity remain unclear. This study on the role of IL-6 and IL-21 during Th17 differentiation and the development of experimental arthritis revealed synergistic effects of IL-6 and IL-21 signaling pathways in driving Th17 differentiation *in vitro*, and indicated that the IL-6/IL-21 combination blocking strategy may be a highly effective way of treating patients with early rheumatoid arthritis.

In accordance with previously reported data on *in vitro* Th17-differentiation [17,18], we showed significantly lower levels of IL-17-producing CD4+ cells when differentiating naïve T cells in the absence of IL-6. However, we observed no reduction in Th17 differentiation when solely IL-21 signaling was blocked, indicating that IL-21 is not a critical factor during Th17 differentiation in vitro. Data from earlier studies on the role of IL-21 in in vitro Th17 differentiation are ambivalent. IL-21 is suggested to be essential for differentiation of Th17 cells [20], whereas others claim IL-21 is capable of, but not necessary for, driving Th17 development [31,32], the latter being in line with the current study. Although we cannot fully explain this discrepancy, the reason might lie within the concentrations of the various cytokines and antibodies used to drive T cell differentiation, or within the mouse strain used to isolate naïve T cells. Interestingly, when both IL-6 and IL-21 pathways were inactive, differentiation of naïve T cells towards Th17 cells was almost completely blocked, clearly showing the synergy between the two pathways in driving Th17 differentiation *in vitro*.

Accordingly, significantly reduced Th17 levels were present in draining lymph nodes of arthritic mice in which the IL-6 pathway was blocked, two days after induction of the AIA model. Moreover, blocking both IL-6 and IL-21 pathways resulted in almost undetectable Th17 levels, showing the importance of these two cytokines in driving Th17 differentiation *in vivo*. As IL-6 and IL-21 are both important in inducing IL-23 receptor expression in naïve CD4+ T cells, this combined blockade might reduce IL-23R expression levels, and thereby IL-23 signaling, to a minimum, possibly contributing to the major reduction in Th17 levels [20,22], In line with previously published data, arthritis severity was significantly reduced in IL-6^-/-^ mice as compared to WT controls, two days after arthritis induction [33]. Earlier work of our group showed reduced arthritis severity in IL-21R^-/-^ mice as compared to WT controls [24], the present study confirms these data. Additionally, both studies show reduced IL-17 production by CD4+ cells of mice lacking expression of the IL-21 receptor. Interestingly, as opposed to this T cell effect, previous studies showed the reducing effect of IL-21 blockade on arthritis development could be attributed to a defect in B cell development rather than in T cell development in the K/BxN and collagen-induced arthritis (CIA) murine arthritis models [34,35]. In contrast to our study, Th17 responses were unaffected in IL-21R^-/-^ mice with CIA [35], as well as in IL-21R^-/-^ mice with experimental autoimmune encephalitis (EAE) and myocarditis (EAM) [32]. The exact reason for this discrepancy in mechanism of disease reduction remains unclear, but might reflect the pleiotropic character of IL-21, with the genetic and inflammatory context influencing the functioning of the cytokine. Regardless of the mechanism of action, a block in IL-21 reduced experimental arthritis development in the majority of reported studies, including the present study. Of high interest, our double blocking strategy was most effective in reducing arthritis severity, which is in concordance with Th17 levels of those animals. These data hint towards therapeutic potential of using a combined IL-6/IL-21-blocking treatment strategy.

Indeed, when treating mice with both anti-IL-6R antibodies and sIL-21R.Fc either during the induction phase of arthritis (day 0) or in a later stage of the disease (day 21), arthritis incidence levels could potently be reduced, confirming the therapeutic potency of this strategy in a RA mouse model. Also in comparison with mice treated solely with anti-IL-6R antibodies, a compound currently used to effectively treat RA patients [36] and known as Tocilizumab (JW Pharmaceutical, Seoul, South Korea), the combination treatment proved to be much more potent in preventing arthritis development. This holds true when treatment was initiated early during arthritis development, but especially when first therapeutic injections were administered in a later stage of the disease, where the anti-IL-6R antibodies could not prevent disease onset. Tocilizumab is a humanized anti-IL-6R monoclonal antibody that has been tested in the clinic both as a monotherapy, as well as in combination with disease-modifying anti-rheumatic drugs (DMARDS), the first-line treatment for RA patients. Tocilizumab monotherapy potently inhibited structural joint damage, and led to increased percentages of patients achieving ACR20, 50 and 70 responses. Additionally, the number of patients achieving remission as defined by a Disease Activity Score in 28 Joints (DAS28) lower than 2.6, was increased in Tocilizumab-treated patients [37–40]. Samson et al. suggest that the successes of Tocilizumab may be attributed to a correction of the imbalance between the pathogenic Th17 cells and the protective regulatory T (Treg) cells that is found in part of the RA patients [41]. They showed decreased Th17 levels in peripheral blood of RA patients after Tocilizumab treatment, whereas Treg numbers were increased. As arthritis development in mice was more effectively reduced by our combination treatment than by the anti-IL-6R antibodies, a similar response may be expected when treating patients with Th17-driven joint pathologies, suggesting the potential of adding an anti-IL-21 treatment to the currently used Tocilizumab monotherapies.

In addition to reduced incidence after anti-IL-6R/anti-IL-21 treatment, disease severity of arthritic mice receiving early combination therapy was reduced. Interestingly, neither anti-IL-6R antibodies, nor anti-IL-21 therapy as single treatments could reduce arthritis severity in the severe and progressive collagen-induced arthritis model. A partial difference in the outcome of anti-IL-6R antibody treatment is present compared to earlier studies [27,42], which may be due to different study design. In line with our data, no protective effect was observed when this treatment was initiated later in disease development. Radiological bone damage of the knee and ankle joints was significantly reduced only in mice receiving early treatment with anti-IL-6R antibodies alone, a trend towards reduction was observed when combined with anti-IL-21 therapy, to levels comparable to that of anti-TNFα treated mice. Histological damage was only reduced by the combination treatment, again to levels comparable to those observed in mice treated with TNFα inhibitors.

Interestingly, a clear trend towards reduced Th17 levels in draining lymph nodes of mice receiving either early single or combination treatment was present, despite the late time point of analysis (day 35). In contrast, no reduced Th17 levels were observed when treatment was initiated later in disease development. Data of mice treated with anti-IL-6R antibodies initiated at both time points confirm previously published data [42]. Although our *in vitro* data demonstrated synergy between IL-6 and IL-21 in Th17 differentiation, this was not clearly reflected by Th17 levels in draining lymph nodes of arthritic mice during our *in vivo* studies. Determination of T cell levels in inflamed synovium may provide more information on local differences in T cell subsets.

Over the past few years, clinical trials have been performed testing various IL-17-targeting agents for a number of autoimmune diseases, amongst others Rheumatoid Arthritis. In RA patients who failed to respond to synthetic and/or biologic DMARDs, both ixekizumab and secukinumab, two agents targeting the IL-17A ligand, induced small but clinically relevant responses. Reported safety profiles were similar to that of other biological agents [43–48]. In contrast, no clinical response was observed when treating RA patients with the anti-IL-17-receptor antibody brodalumab [49,50], a clear explanation for the differences in efficacy of ligand versus receptor targeting is lacking. Even though these studies indicate targeted inhibition of the IL-17A ligand may have therapeutic value in the treatment of RA, pharmaceutical companies appear to shift the application of their anti-IL-17 therapies to other rheumatic diseases like psoriatic arthritis [51,52] and ankylosing spondylitis [53]. Most likely because obtained clinical effects of IL-17 blocking strategies in RA were below expectation, especially when comparing with the impressive therapy responses observed in patients suffering from plaque psoriasis [54].

As IL-17 and other Th17-derived cytokines are important contributors to RA pathology [8–16], identifying other ways to target this pathway remains a high priority. With this study we demonstrated that full inhibition of both IL-6 and IL-21 effectively abrogates Th17 differentiation, and that, despite the minor effect on Th17 development, a combination therapy neutralizing both cytokine pathways is more effective in treating early T cell-driven experimental arthritis than targeting either cytokine. This indicates that anti-IL-6/IL-21 combination therapy might be an interesting new strategy to treat early RA patients or to sustain RA remission.

## Supporting information

S1 Table Supporting data Figs 1–6. (PDF)
