# Supplementary material for: Higher efficacy of anti-IL-6/IL-21 combination therapy compared to monotherapy in the induction phase of Th17-driven experimental arthritis
Source: PLoS One. 2017 Feb 3;12(2):e0171757. doi: 10.1371/journal.pone.0171757 (PMC5291406; doi:10.1371/journal.pone.0171757)
Supplement: S1 Table — (PDF) [file pone.0171757.s001.pdf]

S1 Table. Supporting data Figures 1-6.

Figure 1

Fig 1B. % IL-17+ of CD4+

WT + IL-6	WT - IL-6	IL-21R$^{-/-}$ + IL-6	IL-21R$^{-/-}$ - IL-6
98	62,8	14,7	2,75
99,9	75,6	81,7	3,69
93,2	70	90,4	2,26
33,9	19	93,6	1,81
45,8	24,2	71,6	7,37
99,9	19,4	83,9	83,8
98,6	20,6	94,1	
98,6	37,6	96,1	
98,8	53		
98,4	80,5		

Fig 1C . IL-17 level (pg/ml)

WT + IL-6	WT - IL-6	IL-21R$^{-/-}$ + IL-6	IL-21R$^{-/-}$ - IL-6
4563,035	3328,92	4032,545	1999,51
4064,135	3339,755	4180,545	2059,435
4234,12	3386,285	4408,975	1571,715
3718,895	2905,245	4742,21	1361,53
4718,675	3616,67	4235,83	2856,7
4884,9	3211,45	4562,36	3274,32
4874,445	3345,555	4382,935	
4201,505	2975,965	4854,315	
4415,79	3180,965		
4692,82	3037,315		

Fig 1D . IL-21 level (pg/ml)

WT + IL-6	WT - IL-6	IL-21R$^{-/-}$ + IL-6	IL-21R$^{-/-}$ - IL-6
3499,105	1,33	369,065	0,915
4988,15	2,15	5689,495	1,33
3573,415	1,33	3378,84	3,01
2281,225	OOR<	6461,205	0,51
2669,105	1,33	8265,615	OOR<
4714,65	2,58	5294,59	2,15
4271,87	2,19	8825,035	
456,12	OOR<	6073,625	
3316,44	2,15		
4223,53	0,915		

OOR< Out of Range Below

Fig 1E . IL-22 level (pg/ml)

WT + IL-6	WT - IL-6	IL-21R$^{-/-}$ + IL-6	IL-21R$^{-/-}$ - IL-6
669,93	7,805	664,795	4,805
627,995	7,5	315,84	3,255
650,07	7,555	381,035	3,77
678,035	12,54	390,12	3,275
696,795	13,225	341,975	14,27
709,865	11,455	397,415	7,555
590,75	11,685	256,38	
636,48	6,035	571,915	
633,595	13,66		
607,945	13,835		

Figure 2

Fig 2A. % IL-17+ of CD4+

WT	IL-6$^{-/-}$	IL-21R$^{-/-}$	IL-6$^{-/-}$ x IL-21R$^{-/-}$
0,36	0,13	0,2	0,07
0,26	0,12	0,19	0,05
0,23	0,11	0,19	0,07
0,17	0,12	0,16	0,05
0,23	0,06	0,17	0,03
0,27	0,08	0,12	0,02
0,3	0,07	0,09	0,05
0,27	0,07	0,07	0,07
0,29	0,07	0,2	0,07
0,33	0,11	0,21	0,04

Fig 2B. Antibody titer (OD/50)

	WT	IL-6$^{-/-}$	IL-21R$^{-/-}$	IL-6$^{-/-}$ x IL-21R$^{-/-}$
**IgG1**	33350	4542	11734	199,6
	10328	4947	1803	140,9
	11734	3543	390,2	OOR<
	6572	948	279,7	550
	8944	1960	206,4	33,48
**IgG2b**	12820	701	18177	OOR<
	18778	375,4	439,5	56,72
	2537	394,9	119,5	OOR<
	10572	353	166,7	OOR<
	1814	550,4	74,63	47,88
**IgG Total**	OOR>	5018	OOR<	30,66
	OOR>	2588	5236	4107
	98052	3793	15,3	OOR<
	OOR>	2081	1002	6175
	5935	4293	249,6	315,8

OOR< Out of Range Below

Figure 3

Fig 3A. Joint swelling day 1 (R/L ratio)

WT	IL-6$^{-/-}$	IL-21R$^{-/-}$	IL-6$^{-/-}$ x IL-21R$^{-/-}$
1,74	1,6	1,52	1,22
1,66	1,6	1,51	1,5
1,56	1,74	1,54	1,44
1,73	1,52	1,47	1,19
1,75	1,6	1,55	1,31
2	1,68	1,41	1,29

Fig 3B. Joint swelling day 2 (R/L ratio)

WT	IL-6$^{-/-}$	IL-21R$^{-/-}$	IL-6$^{-/-}$ x IL-21R$^{-/-}$
1,5	1,29	1,34	1,25
1,63	1,3	1,32	1,16
1,37	1,32	1,28	1,15
1,61	1,38	1,25	1,22
1,55	1,4	1,33	1,17
1,64	1,28	1,38	1,24

Fig 3C. Joint swelling day 4 (R/L ratio)

WT	IL-6$^{-/-}$	IL-21R$^{-/-}$	IL-6$^{-/-}$ x IL-21R$^{-/-}$
1,25	1,02	0,98	1,07
1,13	1,03	1,06	0,92
1,25	1,13	1,08	1,05
1,18	1,08	1,02	1,06
1,25	1	0,95	1,05
1,32	0,98	1,06	1,01

Fig 3D. Histological score (0-3)

	WT	IL-6$^{-/-}$	IL-21R$^{-/-}$	IL-6$^{-/-}$ x IL-21R$^{-/-}$
**Inflammation**	1,5	0,5	0,25	0,25
	2	0,75	1	0,5
	0,75	0,25	1,75	0,25
	2,25	0,5	0,5	0
	1,75	1	0,25	0,25
	0,75	0,75	0,5	0,5
	1,25	0,5	0,25	0
	1,25	0,5	0,5	0,5
	1,75	0,5	0,5	0,25
	2	0,75	0,5	0,5
**PG Depletion**	3	0,75	0,5	0
	3	2	2,25	0,25
	2,75	0,75	2,75	0
	2,75	0,5	0,5	0,25
	2,75	2,25	0,5	0,5
	2,5	1,25	0,5	0,25
	2,75	1	0,25	1,25
	2,75	1,25	1	1
	2,75	0,75	0,5	0,25
	2,75	0,5	0,25	0,5
**Bone erosion**	1,487692	1,308462	1,103077	1,282308
	1,769231	1,281538	1,358462	1,307692
	1,357692	1,308462	1,717692	1,359231
	1,897692	1,153846	1,307692	1,230769
	1,744615	1,179231	1,307692	
	1,41	1,178462	1,205385	1,359231
	1,281538	1,153846	1,026154	1,102308
	1,359231	1,283077	1,076923	1,281538
	1,691538	1,333077	1,356923	1,102308
	1,82	1,050769	1,204615	1,409231

Figure 4

Fig 4A. % IL-17+ of CD3+CD4+

	Rat IgG1	α-IL-6R Ab	sIL-21R.Fc	Combination
**Early treatment**	2	0,54	1,13	1,67
	4,1	1,14	0,91	1,08
	1,45	1,46	0,99	1,2
	1,87	1,13	1,12	1,03
	2,09	1,06	0,72	1,05
**Late treatment**	1,04	0,64	0,94	0,83
	0,74	0,93	0,88	2,74
	1,36	0,8	1,6	0,71
	1,5	0,89	0,95	1,56
	0,67	0,99	0,64	0,62

Fig 4B. % IFNy+ of CD3+CD4+

	Rat IgG1	α-IL-6R Ab	sIL-21R.Fc	Combination
**Early treatment**	4,6	2,12	5,4	10,6
	9,67	4,48	7,71	7,5
	6,35	7,54	0	8,89
	4,6	2,45	5,17	6,76
	5,49	8,56	7,16	15,1
**Late treatment**	8,02	11,8	8,7	4,89
	9,24	6,79	8,13	9,12
	3,34	3,79	6,19	4,84
	6,25	6,71	6,19	8,16
	5,19	6,25	4,04	3,53

Fig 4C. CII Antibody titer (OD/50)

	Rat IgG1	TNFα inhibitor	α-IL-6R Ab	sIL-21R.Fc	Combination
**IgG1**	299,1	1215	2429	577	453,8
	508,2	437,5	143,4	1699	272,1
	1544	102,5	3007	723,9	139,6
	274,3	104	46,38	950,1	69,06
	67,03	70,18	29,04	48,26	135,8
**IgG2a**	759	305,6	2670	394	308,3
	358,9	221,6	160,8	1245	246,5
	772,4	21,47	243,9	895,1	177,5
	98,41	30,36	155,6	625,8	52,65
	37,7	109	105,7	206	224,2
**IgG Total**	548,7	318,7	2879	553,8	267
	428,7	342,4	72,4	2258	351,4
	1517	30,17	855,8	804,5	114,9
	198,9	18,16	89,3	842,3	23,4
	36,13	63,61	37,1	208,5	77,71

Figure 5

Fig 5A. Arthritis incidence (%)

Days after immunization	Rat IgG1	TNFα inhibitor	α-IL-6R Ab	sIL-21R.Fc	Combination
**16**	20	0	0	20	0
**18**	40	0	0	40	0
**21**	60	0	0	60	0
**23**	60	0	0	80	0
**25**	80	0	0	80	0
**28**	100	0	40	80	0
**30**	100	20	60	100	20
**32**	100	20	60	100	20
**35**	100	40	60	100	40

Fig 5B. Macroscopic score (0-8)

Rat IgG1	TNFα inhibitor	α-IL-6R Ab	sIL-21R.Fc	Combination
3,75	2,25	5,75	3,5	2
3,75	0,25	2,5	4,75	0,75
4	0	1,5	5,25	0
2,75	0	0	4	0
1,75	0	0	3	0

Fig 5C. Radiological damage (0-8)

Rat IgG1	TNFα inhibitor	α-IL-6R Ab	sIL-21R.Fc	Combination
0,75	1	1,75	2,5	1,75
5	0,75	1	3,5	0
3,5	0,5	0	4	0,25
2,25	0	0,25	4	0
1,75	0	0	0	0,25

Fig 5D. Histological score (0-3)

	Rat IgG1	TNFα inhibitor	α-IL-6R Ab	sIL-21R.Fc	Combination
**Inflammation**	0,75	0,5	2,25	2,25	0,25
	2,25	0,5	2,75	2,25	2,25
	2,75	0	0,25	2,75	0,25
	2	0,25	0,75	1,25	0,25
	1	0	0,75	2	0
	1,75	0,25	0,25	2	0
	2,75	0	0,25	2,5	0
	0,5	0	0	2,75	0
	1,5	0	0,25	0,25	0
	0,25	0	0,25	1	0,25
**PG Depletion**	0,75	0,5	2,5	2,5	0
	2,5	0	2,75	2,5	2,5
	2,75	0	0	2,75	0
	3	0	1,25	3	0
	2,25	0	0,625	3	0
	3	0	0	3	0
	2,75	0	0	3	0
	0	0	0	2,75	0
	1	0	0	0	0
	0	0	0	1	0
**Bone erosion**	0,81	0,58	0,67	1,19	0,44
	0,79	0,56	1,75	1,38	1,21
	2	0,69	0,56	1,71	0,29
	2,46	0,75	0,88	1,67	0,71
	0,48	0,63	0,52	1,46	0,69
	1,88	0,63	0,69	2,29	0,58
	1,67	0,31	0,82	2,42	0,63
	0,38	0,42	0,85	1,83	0,88
	1,08	0,9	1,06	0,75	0,67
	0,67	0,5	0,54	0,56	0,46

Figure 6

Fig 6A. Arthritis incidence (%)

Days after immunization	Rat IgG1	TNFα inhibitor	α-IL-6R Ab	sIL-21R.Fc	Combination
**16**	0	0	0	0	0
**18**	20	0	0	0	0
**21**	60	20	20	40	20
**23**	100	40	100	100	60
**25**	100	60	100	100	60
**28**	100	60	100	100	60
**30**	100	60	100	100	60

Fig 6B. Macroscopic score (0-8)

Rat IgG1	TNFα inhibitor	α-IL-6R Ab	sIL-21R.Fc	Combination
1,5	2	5,5	4	5,5
3,75	3,75	2,5	6	7,25
3	1,5	2,25	5,5	4,5
4,25	0	4,5	7,25	0
2	0	3,5	5,5	0

Fig 6C. Radiological damage (0-8)

Rat IgG1	TNFα inhibitor	α-IL-6R Ab	sIL-21R.Fc	Combination
0,75	0,5	3,5	3	2,25
2	1	1	2,25	3,75
3,25	0,5	1,5	2,25	2
1,75	0,5	2,75	3,75	1,25
2	0,5	2,75	2,25	0

Fig 6D. Histological score (0-3)

	Rat IgG1	TNFα inhibitor	α-IL-6R Ab	sIL-21R.Fc	Combination
**Inflammation**	1	0	1,75	2,5	1,75
	0,25	0	1,5	2,25	1,25
	0,5	0,25	2	2,25	2,25
	2,25	0	0,25	1,75	2,25
	0,5	0	0,25	2,75	1,5
	3	0	1,5	2,5	0
	2,25	0,25	2,25	2,5	0,25
	0	0,25	0,5	2,25	0
	0	0,25	0,25	1,75	0
	2,25	0,25	2	2,25	0
**PG Depletion**	0,75	0	1,75	2,75	1,75
	0	0	2,5	3	1
	0,25	0	2,25	2,25	2,5
	2,5	0,25	0	2,25	2,5
	0,75	0	0,25	3	1,75
	3	0	2,25	2,5	0
	2,75	0	2,75	2,5	0
	0	0,25	0,5	2,75	0
	0	0	0	2	0
	2,75	0	2,25	2,75	0,25
**Bone erosion**	0,71	0,46	1,21	1,33	1
	0,38	0,72	0,81	1,63	1,54
	0,38	0,79	1,79	1,67	1,5
	1,38	0,79	0,71	1,5	1,63
	0,75	0,46	0,54	1,67	1,5
	2,38	0,75	1,17	1,42	0,54
	1,88	0,46	1,67	1,79	0,56
	0,92	0,58	0,46	1,29	0,42
	0,75	0,58	0,58	0,92	0,54
	1,63	0,29	1,17	1,42	0,33
